# Deworming among preschool age children in sub-Saharan Africa: pooled prevalence and multi-level analysis

**DOI:** 10.1186/s41182-022-00465-w

**Published:** 2022-10-08

**Authors:** Daniel Gashaneh Belay, Anteneh Ayelign Kibret, Mengistie Diress, Yibeltal Yismaw Gela, Deresse Sinamaw, Wudneh Simegn, Amare Agmas Andualem, Abdulwase Mohammed Seid, Desalegn Anmut Bitew, Mohammed Abdu Seid, Habitu Birhan Eshetu, Tsega Degu Jemere, Yalelet Fentaw Shiferaw, Yadelew Yimer Shibabaw, Dagmawi Chilot

**Affiliations:** 1 Department of Human Anatomy, School of Medicine, College of Medicine and Health Science, University of Gondar, Gondar, Ethiopia; 2 Department of Epidemiology & Biostatistics, College of Medicine and Health Science, Institute of Public Health, University of Gondar, Gondar, Ethiopia; 3 Center for Innovative Drug Development and Therapeutic Trials for Africa (CDT-Africa), College of Health Sciences, Addis Ababa University, Addis Ababa, Ethiopia; 4 Department of Human Physiology, School of Medicine, College of Medicine and Health Science, University of Gondar, Gondar, Ethiopia; 5 Department of Biomedical Science, Debre Markos University, Debre Markos, Ethiopia; 6 Department of Social and Administrative Pharmacy, College of Medicine and Health Science, University of Gondar, Gondar, Ethiopia; 7 Department of Anesthesia, Wollo University, Dessie, Ethiopia; 8 Department of Clinical Pharmacy, College of Medicine and Health Science, University of Gondar, Gondar, Ethiopia; 9 Department of Reproductive Health, University of Gondar, Gondar, Ethiopia; 10 Unit of Human Physiology, Department of Biomedical Science, College of Health Sciences, Debre Tabor University, Debra Tabor, Ethiopia; 11 Department of Health Education and Behavioral Sciences, University of Gondar, Gondar, Ethiopia; 12 Department of Nutritional Care and Counseling, University of Gondar Specialized Hospital, Gondar, Ethiopia; 13 Department of Biochemistry, School of Medicine, College of Medicine and Health Science, University of Gondar, Gondar, Ethiopia

**Keywords:** Deworming, Preschool age, Sub-Saharan

## Abstract

**Background:**

Sub-Saharan Africa (SSA) preschool age children are more vulnerable to soil-transmitted helminths (STH) which caused millions of morbidity because of low socioeconomic status and lack of clean water and sanitation. Despite this problem, there is minimal evidence on the prevalence and factors associated with deworming medication utilization among preschool age children (pre-SAC) in SSA regions. Hence this study aimed to assess the prevalence and determinants of deworming among preschool age children in SSA.

**Methods:**

Demographic and Health Survey (DHS) data were used for this study with a total weighted 192,652 children aged 24–59 months. Taking deworming medication in the 6 months preceding the interview was our outcome of interest. A multi-level binary logistic regression model was fitted. Adjusted odds ratio (AOR) with 95% confidence interval (CI) was taken to identify significant variables.

**Results:**

The prevalence of deworming medication utilization among preschool age children in SSA was 45.03% (95% CI 44.46%, 45.60%), ranging from 41.82% in Malawi to 50.5% in Lesotho. It was 44.91% (95% CI 44.32%, 45.51%) among countries having endemic STH infection and 46.01% (95% CI 43.64%, 48.38%) for none endemic countries. Factors such as; secondary and above women education [AOR = 2.18; 95% CI 2.10, 2.26], occupation [AOR = 1.31; 95% CI 1.27, 1.34], having ≥ 11 family members [AOR = 0.68; 95% CI 0.64, 0.70], household media exposure [AOR = 1.16; 95% CI 1.13, 1.19] and richer wealth status [AOR = 1.23; 95% CI 1.16, 1.27], vitamin A supplementation [AOR = 6.18; 95% CI 6.02, 6.33] and living rural residence [AOR = 0.94; 95% CI 0.92, 0.98] have significantly associated with deworming among preschool age children.

**Conclusions:**

Utilization of deworming medication among pre-SAC children in sub-Saharan Africa is below half. Factors, such as the education status of women, family size, household media exposure, wealth status, diarrhea, vitamin A supplementation, and residence were significant variables. To increase the utilization of deworming medication for pre-SAC, WHO should work as an integrated approach with other stakeholders, by strengthening women’s education, and media exposure. Maternal employment should be promoted and prior attention should be given to rural children.

## Background

Soil-transmitted helminths (STH) affect nearly two billion people worldwide, 90% of whom are living in sub-Saharan Africa [1–3]. The disease affects people living in rural or deprived urban settings with low socioeconomic status, poor sanitation, and a lack of clean water [2, 3]. Moreover, since they usually play in fecal contaminated soil and their weak immunity, preschool and school-aged children are the most vulnerable group and harbor the greatest numbers of intestinal worms [2, 4–6].

This disease caused millions of morbidity among under-five children who live in developing countries [7]. They experience stunting, and diminished physical fitness as well as impaired memory and cognition [2, 4]. The social and economic consequences of helminthic infections go far beyond the obvious health impacts, including lost school attendance and productive working time [6].

It is recommended to treat all preschool age children with deworming drugs, with 6-month intervals in areas where helminth infection is endemic [8]. The strategic plan was to eliminate STH as a public health problem in children by 2020 [9]. Annual or biannual preventive chemotherapy (deworming), using a single dose of albendazole or mebendazole is recommended as a public health intervention for all young children 12–23 months of age, preschool children 2–5 years of age, and school-age children 6–12 years of age living in areas where the baseline prevalence of any soil-transmitted infection is 20% or more among children [10]. Periodic STH deworming has been shown to prevent anemia among preschool age children (pre-SAC) by avoiding iron loss ascribed to hookworm infection [11]. It also improves growth [12], and motor and language development in pre-SAC [13]. The delivery of albendazole every 6 months can reduce 28% of diarrhea episodes in pre-SAC [14]**,** reduce stunting by 9.4%, and improve weight by 35% in infants and pre-SAC within 2 years [14, 15].

Global deworming programs aim to reach 75% of at-risk preschool-age children by 2020 [5, 9]. But, between 2004 and 2017 the mean global deworming coverage in pre-SAC was estimated at 36% [16].

Therefore this study aimed to assess the prevalence of deworming medication utilization and to identify the individual and community-level factors associated with it among pre-SAC in the sub-Saharan African countries.

## Methods

### Study design, setting, and period

The data source was the latest standard Demographic and Health Survey (DHS) data set of sub-Saharan African countries within 10 years (2010–2020). To get all parameters and a large sample size which make representative of the source of population, we used the standardized data set [17]. DHS collects data that are comparable across countries. The surveys are nationally representative of each country and population-based with large sample sizes [17].

The sub-Saharan is the area in the continent of Africa that lies south of the Sahara desert and consists of four vast and distinct regions, i.e., Eastern Africa, Central Africa, Western Africa, and Southern Africa. Together, they constitute an area of 9.4 million square miles and a total population of 1.1 billion inhabitants [18].

### Populations

All children aged 6–23 months preceding 5 years of the survey period across 33 sub-Saharan African countries were our source population, whereas children aged 24–59 months in the selected Enumeration Areas (EAs) or primary sampling units of the survey clusters were our study populations. The mother or the caregiver was interviewed for the survey in each country and mothers who had more than one child within the 2 years preceding the survey were asked questions about the most recent child [19]. Moreover, from the included countries’ data set, children in the age category of 24–59 months who are not assessed to deworm medication based on the DHS guideline, and have the missing value of the outcome variable were excluded.

### Sample size determination and sampling method

Of the total of 47 countries located in sub-Saharan Africa, only 41 countries had Demographic and Health Survey Report. Of these, five countries namely; Central Africa Republic (DHS report 1994/95), Eswatini (DHS report 2006/07), Sao Tome Principe (DHS report 2008/09), Madagascar (DHS report 2008/09), and Sudan (DHS report 1989–90) have a survey report before the 2010 survey year and excluded from further analysis. Moreover, three countries (Botswana, Mauritania, and Eritrea) were excluded due to the DHS dataset not being publicly available. Finally, a total of 33 sub-Saharan African countries were represented in this study in the four regions namely Eastern Africa, (Burundi, Comoros, Ethiopia, Kenya, Malawi, Mozambique, Rwanda, Tanzania, Uganda, Zambia, Zimbabwe), Central Africa (Angola, Cameroon, Chad, the Democratic Republic of the Congo, Republic of the Congo, Gabon), Western Africa (Benin, Burkina Faso, Ivory Coast, Gambia, Ghana, Guinea, Liberia, Mali, Niger, Nigeria, Senegal, Sierra Leone, Togo) and Southern Africa (Lesotho, Namibia, South Africa) [20]. Of these, only five countries had not the endemic prevalence of STH infections based on WHO reports [9].

In general, the most recent standard census frame was used in all of the surveys conducted in the selected countries. Typically, DHS samples are stratified by administrative geographic region and by urban/rural areas within each region. In the first stage of sampling, enumeration areas (EAs) were selected with probability proportional to size within each stratum. In selected EAs, a fixed number of households is selected by the systematic sampling method in the second stage of sampling. Following the listing of the households, a fixed number of households is selected by equal probability systematic sampling in the selected cluster [17].

The children’s records or kid’s records (KR) DHS datasets were used. Weighted values were used before using the DHS dataset to restore the representativeness of the sample data. Since the overall probability of selection of each household is not constant. DHS guidelines set four sampling weighting methods and from that, we used the individual weight for women (v005). Individual sample weights are generated by dividing (v005) by 1,000,000 before being used to approximate the number of cases [21]. Finally, a total weighted sample of 192,652 children in the age category of 24–59 months from all 33 countries was included in this study.

### Study variables

The outcome variable of this study was taking deworming medication by preschool aged children. During the survey, their mother was asked questions about their under 5 years children who took deworming medication in the 6 months preceding the interview, and if they answered ‘yes’ they are considered as taking deworming [17]. Endemicity was categorized as endemic and non-endemic based on the WHO report on STH prevalence [9]. Individual and community-level independent variables have been studied (Table 1).

**Table 1:** Individual and community-level independent variables in the study of deworming medication intake and associated factors among 24- to 59-month-old children in SSA

Level	Variables	Measurements
Individual-level variables	Age	The age of the mother/caregiver is categorized as 15–19, 20–34, and 35–49 [22]
	Sex	Sex of the household head as male or female
	Education level	Educational attainment is categorized as uneducated, primary, secondary, and above educational status
	Marital status	The marital status of the mothers is categorized as married or not married
	Occupation of women	The occupations of women are categorized as working (professional/technical/managerial, clerical sales agricultural, employee, services, skilled manual unskilled manual, others) and not working
	Family size	Categorized as 1–4, 5–10, and 11 and above [22]
	Media exposure	A composite variable was obtained by combining whether a respondent reads newspaper/magazine, listens to the radio, and watches television with a value of “0” if women were not exposed to at least one of the three media, and “1” if a woman has access/exposure to at least one of the three media [23]
	Wealth index	The datasets contained a wealth index that was created using principal components analysis coded as poorest, poorer, middle, richer, and richest in the DHS data set. For this study, we recorded it in three categories poorer (including poorer and poorest), middle and richer (includes richer and richest) [22]
	Sex of the child	The sex of the child is categorized as male or female
	Age of the child	The age of the child is categorized as 24–35, 36–47, and 48–59 months
	Vitamin A	Vitamin A in the last 6 months, categorized as yes or no
	Diarrhea	Diarrhea in 2 weeks, categorized as yes or no
Community-level variables	Residency	Urban or rural based on where the household lives
	Region in SSA	The region in sub-Saharan African region is categorized as Eastern Africa, Central Africa, Western Africa, and Southern Africa
	Countries income level	The country’s income status was categorized as low income, lower middle income, and upper-middle income country based on the World Bank List of Economies classification since 2019 [24]. World Bank calculated country income based on Gross National Income (GNI) per capita, which categorized as low income $1025 or less; lower middle income, $1026–3995, upper-middle income $3996–12,375, and high income $12,375 or more [24]
	DHS survey year	The survey year means the recent standard DHS data collection period of each country from 2010 to 2020. Categorized as DHS year 2010–2012, 2013–2015, and 2016–2020

### Data processing and analysis

The standard DHS dataset was downloaded in STATA format then cleaned, integrated, transformed, and appended to produce favorable variables for the analysis. Microsoft Excel and STATA version 14.2 software [25] were used to generate both descriptive and analytic statistics to describe variables in the study using statistical measurements.

### Model building for multi-level analysis

Since the DHS data have hierarchical nature, children were nested within a cluster that violates the standard logistic regression model assumptions such as the independence and equal variance assumptions, a multi-level binary logistic regression model was fitted. Four models were fitted for multi-level analysis. The first was the null model (Model 1) which contains only the outcome variables. It is used to check the variability of deworming utilization across the cluster. The second (Model 2) and the third (Model 3) multi-level models contain individual-level variables and community-level variables, respectively. In the fourth model (Model 4), both individual and community-level variables were fitted simultaneously with the prevalence of deworming utilization. Model comparisons were done with a standard logistics regression model using the log-likelihood and deviance test and the model with the highest log likelihood and lowest deviance was selected as the best-fitted model. The variance inflation factor (VIF) was used to detect multicollinearity between independent variables. Based on the STATA analysis result, all variables had VIF values less than 10 and the mean VIF value of the final model was 1.50 which shows no multicollinearity. In the fixed effect measure of association, the variable which has a significant association with adjusted odds ratio (AOR) ratios was declared using a p-value of < 0.05 with 95% confidence intervals. The random effect used to measure the variation was estimated using the median odds ratio (MOR), intra class correlation coefficient (ICC), and proportional change in variance (PCV) [26–28].

## Results

### Socio-demographic characteristics of mothers or caregivers

A total weighted sample of 192,652 children of age 24–59 months were included in this study. Nearly half (50.29%) of mothers of children were found in the age group of 20–34 years, with a median age of 29 (IQR: 25, 35) years. More than two-fifths (41.63%) of mothers/caregivers had no formal education. Most of the respondents lived in West Africa and East Africa regions (79.71%) and from rural (68.48%) residences. More than three-fourths (78.7%) of the children have taken vitamin A in the last 6 months (Table 2).

**Table 2:** Socio-demographic characteristics of the mothers/caregivers and the children in a study of deworming among preschool age children in sub-Saharan Africa

Variables	Categories	Weighted frequency (n)	Weighted percentage (%)
Socio-demographic characteristics and health service utilization of the mothers			
Age of women (years)	15–19	46,327	24.05
	20–34	96,885	50.29
	35–49	49,440	25.66
Educational attainment of women	No education	80,195	41.63
	Primary education	59,819	31.05
	Secondary and above	52,639	27.32
Occupation of women	Not working	57,833	30.10
	Worked	134,819	69.90
Household family size	1–4	46,630	24.2
	5–10	119,501	62.03
	≥ 11	26,521	13.77
Media exposure	No	70,703	36.77
	Yes	121,607	63.23
Wealth index	Poorer	84,006	43.61
	Middle	38,596	20.03
	Richer	70,050	36.36
Child related characteristics			
Sex of child	Male	96,867	50.28
	Female	95,785	49.72
Age of child	24–35 months	48,162	33.21
	36–47 months	48,936	33.74
	48–59 months	47,926	33.05
Vitamin A in the last 6 months	No	41,037	21.3
	Yes	151,615	78.7
Diarrhea in 2 weeks	No	168,979	87.71
	Yes	23,673	12.29
Community-level variables			
Income level of the country	Lower	126,468	65.68
	Lower middle	50,948	26.46
	High middle	15,128	7.86
Survey year	< 2015	94,680	49.17
	≥ 2015	97,864	50.83
Residence	Urban	60,537	31.42
	Rural	132,116	68.58
Region in SSA	Central Africa	34,571	17.95
	East Africa	75,313	39.11
	West Africa	78,178	40.6
	Southern Africa	4,483	2.33

### The pooled magnitude of deworming among preschool age children in sub-Saharan Africa

**Fig. 1:**
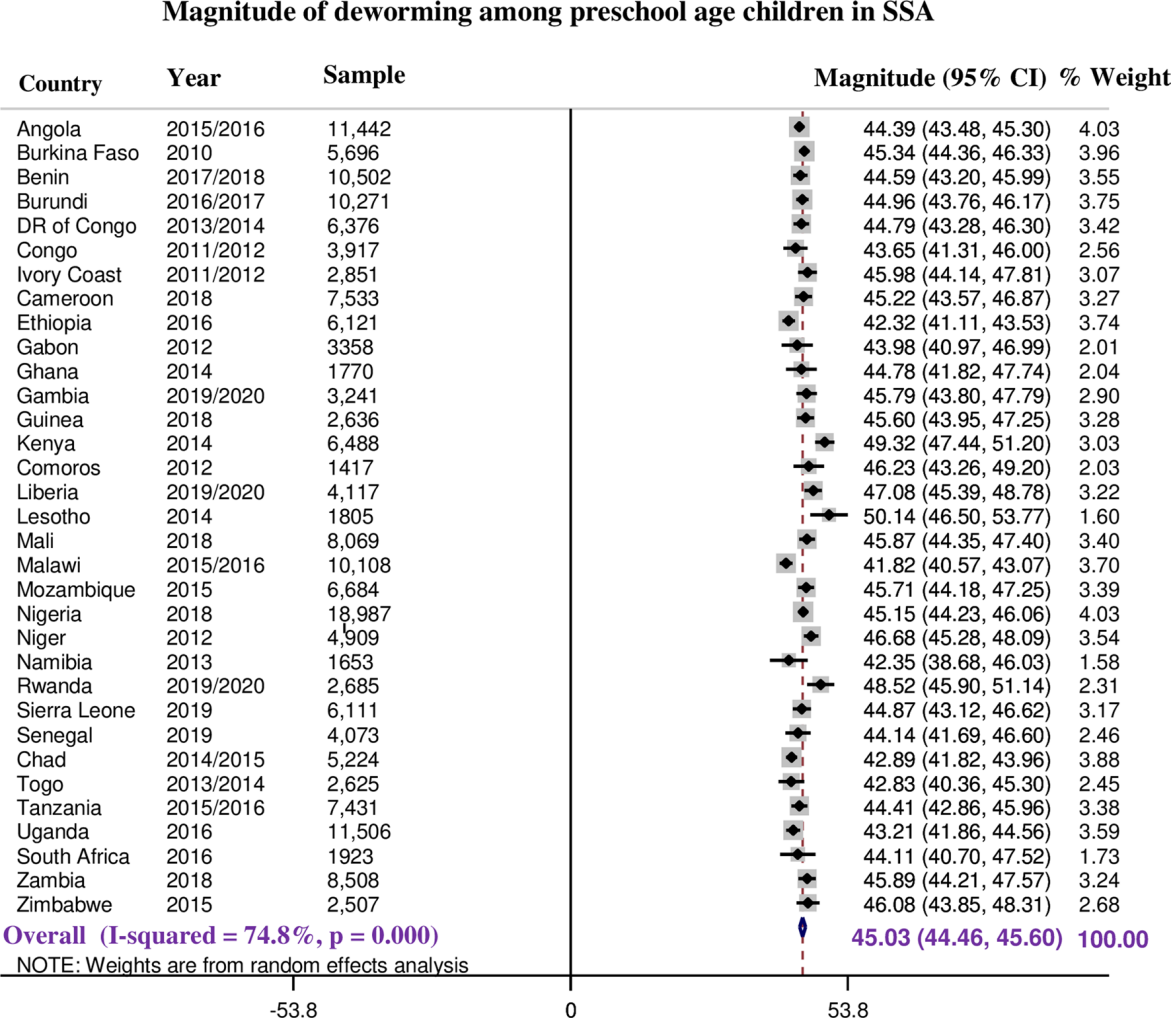
Magnitude of deworming among preschool age children in SSA

**Fig. 2:**
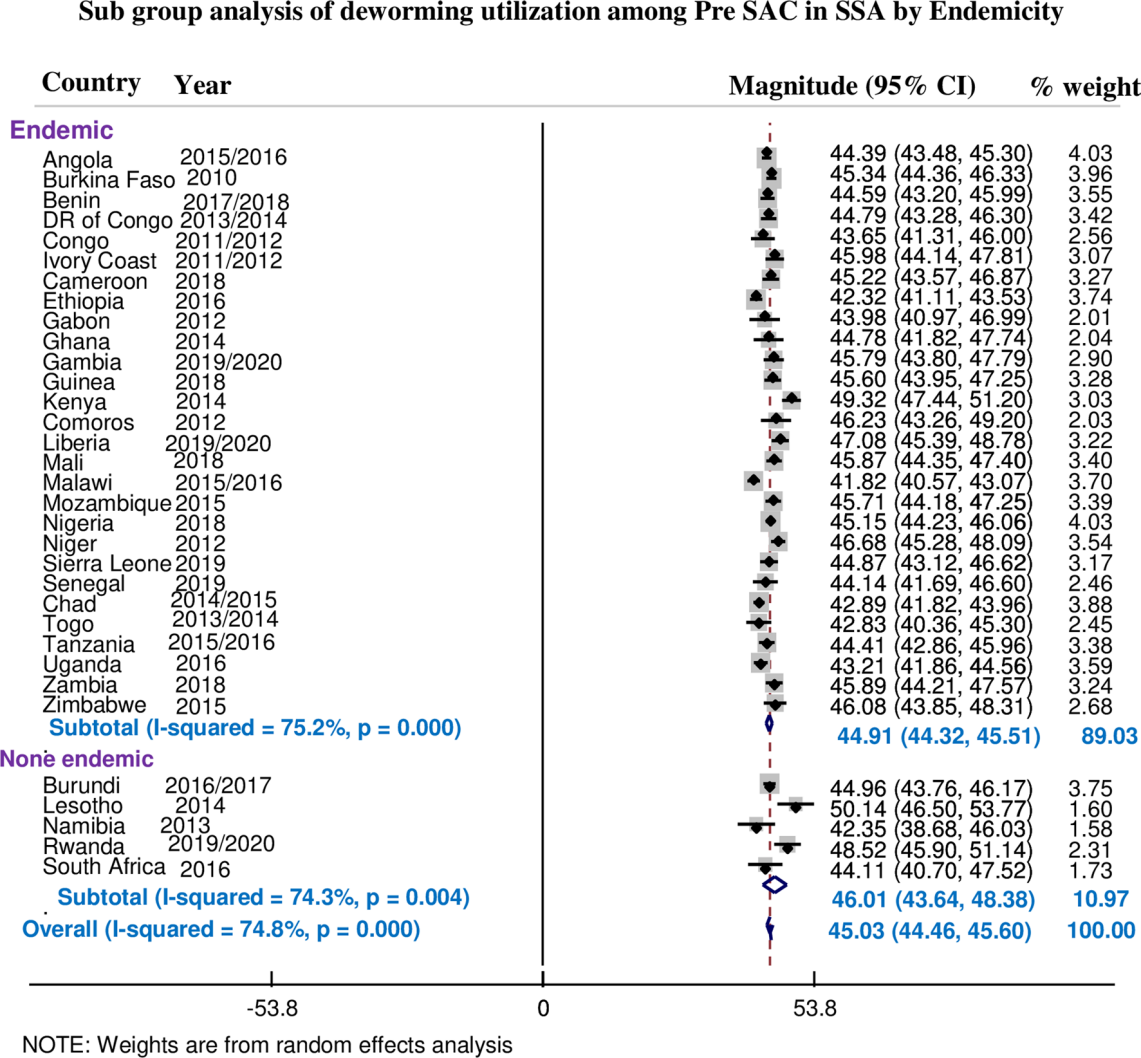
Subgroup analysis of the magnitude of deworming among preschool age children in SSA based on endemicity

The overall pooled estimate of deworming among preschool age children in sub-Saharan African countries was 45.03% (95% CI 44.46%, 45.60%), with *I*^2^ = 74.8% and ranging from 41.82% in Malawi to 50.5% in Lesotho (Fig. 1). The *I*^2^ value shows that there were moderate true variabilities (the variability not by chance) of deworming among preschool age children in 33 countries, then further subgroup analyses were done based on the endemicity. Therefore the prevalence of deworming among 28 SSA countries that have endemic STH infection was 44.91% (95% CI 44.32%, 45.51%) with *I*^2^ = 75.2%, whereas it was 46.01% (95% CI 43.64%, 48.38%) with *I*^2^ = 74.3% among 5 SSA countries which have none endemic STH infection (Fig. 2).

### Multi-level analysis of determinants of deworming among preschool age children in sub-Saharan Africa

#### Model comparison and random effect analysis

Of the four models of multi-level analysis, model 4 is the better model, since it has the lowest deviance value. About 10% of the variations in deworming among preschool children in SSA were attributed to cluster differences. Moreover, about 43.5% of the variation in deworming among preschool children in SSA was explained by both community-level and individual-level variables.

#### Fixed effect analysis

In the final adjusted model of model 4 multi-level logistics regression analysis variables such as; education status of women, media exposure and wealth status of the household, family size, having diarrhea, vitamin A supplementation, and residence had a significant association with taking deworming medication of preschool age children in SSA.

Women who have primary and above primary educational status were 1.95 and 1.18 times more likely to take their child deworming medication than women with no formal education [AOR = 1.95; 95% CI 1.89, 2.00] and [AOR = 2.18; 95% CI 2.10, 2.26], respectively.

The odds of having deworming among preschool age children whose mothers had worked were 1.47 times higher as compared to children from no worked mothers [AOR = 1.31; 95% CI 1.27, 1.34].

As the household family size increase to 5–10 and ≥ 11 members, the odds of deworming preschool children decrease by 11% and 36% [AOR = 0.93; 95% CI 0.89, 0.96] and [AOR = 0.68; 95% CI 0.64, 0.70], respectively.

Children who lived in households that have media exposure and richer wealth status were 16% and 23% more likely to take children deworming medication as compared to households that have no media exposure [AOR = 1.16; 95% CI 1.13, 1.19] and [AOR = 1.23; 95% CI 1.16, 1.27], respectively.

Children who took vitamin A supplementation for 6 months were six times more likely to have deworming medication as compared to those who did not [AOR = 6.18; 95% CI 6.02, 6.33]. Moreover, children who had diarrhea in the last week before the survey were a 16% higher chance of getting deworming medication as compared to no diarrhea episode [AOR = 1.17; 95% CI 1.12, 1.21]. Children who live in rural residence have 6% less likely to take deworming medication as compared to urban [AOR = 0.94; 95% CI 0.92, 0.98] (Table 3).

**Table 3:** Multi-level analysis of factors associated with deworming among children aged 24–59 months in SSA

Variables	Categories	Null model	^a^Model 1 AOR [95% CI]	Model 2 AOR [95% CI]	Model 3 AOR [95% CI]
Age of women (years)	15–19		Ref.		Ref.
	20–35		1.15 [1.11, 1.19]	–	1.15 [1.11, 1.18]*
	36–49		1.31 [1.27, 1.36]	–	1.30 [1.26, 1.35]*
Educational attainment of women	No education		Ref.		Ref.
	Primary education		1.95 [1.89, 2.00]***	–	1.95 [1.89, 2.00]*
	Secondary and above		2.20 [2.12, 2.27]***	–	2.18 [2.10, 2.26]**
Occupation of women	Not worked		Ref.		Ref.
	Worked		1.30 [1.27, 1.35]	_-_	1.31 [1.27, 1.34]***
Household family size	1–4		Ref.		Ref.
	5–10		0.92 [0.90, 0.95]	–	0.93 [0.89, 0.96]*
	≥ 11		0.67 [0.64, 0.70]	–	0.68 [0.64, 0.70]**
Media exposure	No		Ref.		Ref.
	Yes		1.17 [1.14, 1.20]***	–	1.16 [1.13, 1.19]**
Wealth index	Poorer		Ref.		Ref.
	Middle		1.09 [1.05, 1.13]*	–	0.92 [0.71, 1.17]
	Richer		1.26 [1.22, 1.30]***	–	1.23 [1.16, 1.27]*
Sex of child	Male		Ref.		Ref.
	Female		0.98 [0.95, 1.00]	–	0.98 [0.95, 1.01]
Age of child	24–35 months		Ref.		Ref.
	36–47 months		0.99[0.96, 1.02]	–	0.99[0.96, 1.02]
	48–59 months		1.00[0.97,1.03]		1.01[0.97,1.03]
Vitamin A	No		Ref.		Ref.
	Yes		6.16 [6.01, 6.32]***	–	6.18 [6.02, 6.33]***
Diarrhea	No		Ref.		Ref.
	Yes		1.16 [1.13, 1.21]*	–	1.17 [1.12, 1.21]**
*Community-level variables*					
Income level of the country	Lower-income			Ref.	Ref.
	Lower middle		–	1.02 [1.55, 1.04]	0.95[0.92,0.99]
	Upper middle		–	0.99[0.94,1.04]	1.95 [0.88, 1.02]
Survey year	< 2015			Ref.	Ref.
	≥ 2015		–	0.97 [0.95, 0.99]	0.99[0.96, 1.03]
Residence	Urban			Ref.	Ref.
	Rural		–	0.66 [0.64, 0.67]*	0.94 [0.92, 0.98]**
Region	Central Africa			Ref.	Ref.
	East Africa		–	1.02 [0.98,1.06]	0.95 [0.90,1.00]
	West Africa		–	1.03 [0.99, 1.07]	0.97 [0.90, 1.00]
	Southern Africa		–	1.00 [0.93, 1.08]	1.01 [0.92, 1.12]
*Random effect*					
	Variance	0.33	0.24	0.30	0.23
	ICC	0.10	0.07	0.08	0.06
	MOR	1.50	1.26	1.41	1.24
	PCV	Ref.	37.5%	10.0%	43.5%
*Model comparison*					
	Deviance	130,830	80,065	129,948	80,000
	Mean VIF	–	1.50	1.62	1.87

*AOR* adjusted odds ratio, *CI* confidence interval, *ICC* inter cluster correlation coefficient, *MOR* median odds ratio, *PCV* proportional change in variance, *VIF* variance inflation factors

*P value < 0.05

**P value < 0.01

***P value < 0.001

## Discussion

This study aimed to identify factors associated with the utilization of deworming medication as chemoprophylaxis among pre-SAC in sub-Saharan African countries. Based on this, the pooled estimate of deworming medication utilization among preschool age children in sub-Saharan African countries was 45.03% (95% CI 44.46%, 45.60%), ranging from 41.82% in Malawi to 50.5% in Lesotho. It was 44.91% (95% CI 44.32%, 45.51%) among STH infection endemic SSA countries, whereas 46.01% (95% CI 43.64%, 48.38%) among non-endemic countries. This is higher than the schistosomiasis and soil-transmitted helminthiases progress report, 2020 which has 9.21% coverage among 13 African countries [29]. But, our study is lower than a study conducted in 39 countries UNICEF offices (49.1%) [5] and global deworming programs aim to reach 75% pre-SAC by 2020 [5, 9]. These differences might be due to variations in socio-cultural aspects among study participants and due to differences in awareness level and familiarity with the importance of deworming for the prevention of STH infections in preschool children [30, 31]. This data was collected based on standardized similar questionnaires developed Even if there is no significant difference, the higher prevalence of deworming in non-endemic countries might be due to increased health-seeking behavior and adaptations of the deworming program eventually leading to controlling the STH infections. The difference in sample size included in the two groups might be the other variations.

This study results that women who have primary and above primary educational status were more likely to have dewormed children than no formal education. This is in line with studies in Cameroon [26], and Ghana [27]. This shows that when mothers become more educated, they are more likely to utilize the deworming medication for their children and themselves. This might be because the educated mother has health-related information and are better at practicing it [27, 28].

In this study, the odds of taking deworming medication among pre-SAC whose mothers had worked were higher. This is supported by a study conducted in Ghana [27], which showed, that working mothers were more likely to deworm their children relative to not working counterparts. This is because of that, the employed people might have exposure to the importance of supplements and the ability to perches medications [24, 27].

As the household family size increase to 5–10 and > 11 members, the odds of deworming preschool children decrease. This is comparable with a study in Ethiopia [30]. This might be because as family size increases they deserve more attention and need more time. As a result, it might be difficult to fill all the needed care for children [30].

In this study, households which have media exposure were more likely to have dewormed children as compared to households that have no media exposure. It is in line with studies in India [29] and 26 sub-Saharan African countries [30]. This is supported by a study done in Nigeria which showed that utilization of health care can be improved when maternal media exposure increases [25]. Exposure to media could have a tremendous role in increasing awareness and knowledge for mothers and the dissemination of health-related information [30].

Children who lived in households that have richer wealth status were more likely to take their child's deworming medication as compared to households that have poorer households. This is supported by wealthier women being more likely to use deworming drugs than the poorer [32]. This might be linked to the ability to perches deworming medication if not free camping supplementation.

Children who took vitamin A supplementation (VAS) within 6 months were six times more likely to have deworming medication as compared to those who did not. This is maintained by a study in Ethiopia [30]. This might be because VAS and deworming supplements are provided in a campaign integrative way [30].

Moreover, children who had diarrhea in the last week before the survey were a 16% higher chance of getting deworming medication as compared to no diarrhea episode. This is similar to a study in Ethiopia [30]. This might be because, during diarrheal disease treatment, mothers/caregivers might get counseling services regarding deworming supplementation [30]. Moreover, the drug used for the treatment of diarrheal disease and deworming are similar [33].

Children who lived in rural residences were less likely to take deworming medication as compared to urban. This is supported by a study in Ghana [34]. This might be due to the availability and accessibility of health services and deworming programs. Moreover, the difference in socioeconomic inequalities between rural and urban might contributed to this difference [34].

## Strength and limitation

The main strength of this study was the use of large sample data which is weighted by individual weight that makes it representative of each country's levels as well as the sub-Sahara Africa region. But, since the data were secondary data collected cross-sectional, would be prone to recall and social desirability bias. The other limitation is that since all populations in each SSA country/sub-nation would not have the same higher prevalence of soil-transmitted helminths, they might not be populations that need regular deworming. But we cannot get a national report on the prevalence of soil-transmitted helminths in each SSA country/sub-nation in each country’s DHS year.

## Conclusion and recommendation

The prevalence of deworming medication among pre-SAC children in sub-Saharan Africa is below half. Individual-level factors, such as the education status of women, family size, media exposure and wealth status of the household, having diarrhea, and vitamin A supplementation, and community-level variables such as residence were significant variables for utilization of deworming medication among pre-SAC in sub-Saharan Africa.

There are six 2030 global targets for soil-transmitted helminthiases and of which achieving and maintaining the elimination of STH morbidity in pre-school and school-age children is the first one [35]. Therefore, to increase the utilization of deworming medication among pre-SAC in sub-Saharan Africa, WHO should work as an integrated approach with other stakeholders, to strengthen women’s education, household, and media exposure. Maternal employment should be promoted and prior attention should be given to rural children.

## Data Availability

Data are available to publically access from the open databases. It can be accessed from the following website: https://dhsprogram.com/data/dataset_admin/login_main.cfm?CFID=10818526&CFTOKEN=c131014a480fe56-4E0C6B7F-F551-E6B2-50.

## Abbreviations

AOR: Adjusted odds ratio
ANC: Antenatal care
CI: Confidence interval
CSA: Central Statistical Agency
EDHS: Ethiopian Demographic and Health Survey
KR: Kids record
MOH: Ministry of Health
pre-SAC: Pre-school age children
SDG: Sustainable Development Goal
STH: Soil-transmitted helminthic
WHO: World Health Organization
